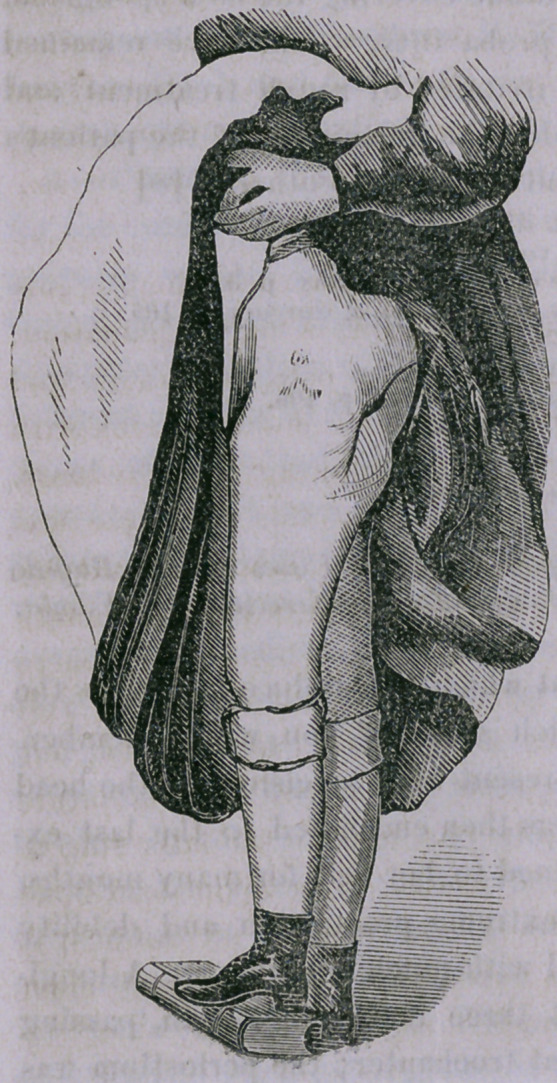# Clinical Remarks upon Surgical Cases in the Buffalo General Hospital—Exsection of Hip-Joint—Exsection of Fibula

**Published:** 1867-02

**Authors:** J. F. Miner


ART. III.—*Clinical Remarks upon Surgical Cases in the Buffalo General Hospital—Exsection of Hip-Joint—Exsection of Fibula.* By J. F. Miner, M. D.

Gentlemen:—The first patient which I introduce to you, is the little girl, Mary Frederick, which some of you will remember. About one year since you were present when excision of the head of the femur was made. She was then emaciated to the last extreme, and had been wholly confined to her bed for many months. Her appearance was that of so extreme prostration and debility that the operation was attempted with much misgiving. A longitudinal incision was made about three inches in length, passing directly over the point of the great trochanter; the periostium was carefully separated from the bone to below the diseased portion, and the head, neck, and trochanters removed, The hemorrhage was exceedingly small, and no ligatures applied. The cavity of the acetabulum was found healthy, but the head of the femur destroyed by the ulcerative processes, which had been actively progressing for two years. The specimen I have preserved with great care, and present it to you; it is a representative one, exceedingly valuable and instructive.

The object this morning is simply to exhibit the diseased bone, and the results which were obtained by the operation, and not to explain its manner or to give other arguments for its justification. Left to themselves, such cases nearly always prove fatal by the slow processes of exhaustion and hectic irritation; to obtain, then, any such result as the one you now observe, is a real triumph of operative surgery. The little girl is restored to health and comfort, to all appearance completely relieved of the disease which seemed certain, if left to itself, to destroy life. The leg is shortened, by removal of the head and trochanters, about two and a half inches, but with the accommodation of the pelvis, common in such cases, this constitutes hardly a perceptible deformity; as you observe she walks nicely without cane or crutch, and with but slight limping. I promised you, at the time of operation, that you should have a report of the result, either verbally or through the pages of the *Buffalo Medical and Surgical Journal.* With the view of better fulfilling my promise, I have obtained a photograph of the present appearance, and will not only show you the result, but will report and represent it for others. I am convinced that many, somewhat similar cases, should be allowed the chances which such an operation offers, and that too frequently they are allowed to progress to a fatal termination, when well directed effort would result in recovery.

You would like to know how the upper end of the bone forms connection with the acetabulum, so that locomotion is attainable. The provisions of nature are truly remarkable, but in few instances is it more manifest and wonderful than in cases like this. The end of the bone heals over and becomes round and smooth; to this rounded end is attached an artificial ligamentum teres, which is joined strongly to the acetabulum or to the rim of the cavity. This ligament is unlike in most respects the natural ligament which unites the head of the femur to the cavity of the acetabulum, but it is dense and strong, and though admitting of great freedom of motion, it still supplies the place of a more perfect connection, and restores, with but little deformity, the natural functions and uses of the joint. The muscular and fibrous structures around the joint also take on new functions and aid in sustaining the bone in its proper position.

*Exsection of the lower end of the Fibula.*—The case which is presented for operation requires a little description and explanation. You will observe that this young man looks pale, emaciated, and very sick. About one year since he became suddenly sick with chills, loss of appetite, strength and flesh, suffering from redness, swelling, and great pain in the lower part of this leg. He was treated for some time for rheumatism by the family attendant, until, at length, pus formed, and was discharged in profuse quantities from various openings. Caries of the bone commences with swelling, redness, fever, and great pain; and at first, if you fail to detect its true nature, you may hope to be excused, but experience and observation will enable you to discover its nature quite early, while any mistake as to its character in the later stages, is wholly unpardonable. The probe passes down upon dead bone over the lower portion of the fibula, and there is no doubt it is extensively diseased. The pain is unbearable, and something must be immediately attempted for its radical relief.

Exsection of the fibula is attended by serious objections; but, however this may be, it seems the only means of affording relief. This bone is said to have been removed in its whole length, and a useful leg still left. It is a matter of some question which is best, amputation or exsection, but it is proper to try the results of the former; the latter can be adopted when no choice remains.

We have now removed the lower half of the fibula, which separates from the periostium easily, and is removed without great disburbance of the parts about the ankle joint; nature seems to have commenced, in its way, the same operation, and we have only assisted, facilitating the result. The condition of our patient can hardly be made worse; if it change very much, it must improve. The pain, irritation and purulent drain, have so exhausted the vital forces that much less confidence is felt in the utility of this operation than if made before this condition of debility had reached this extreme point; however, his age, natural vigor, and strong hope are favorable, and, I trust, we may be able to present him at some future time, another illustration of the value and superiority of well selected conservatism in operative surgery.

## Figures and Tables

**Figure f1:**